# Apomorphine Subcutaneous Infusion Likely Induced Acute Thrombocytopenia in a Patient with Parkinson's Disease and Motor Fluctuations

**DOI:** 10.1002/mdc3.14216

**Published:** 2024-09-27

**Authors:** Maria Magdalena Pocora, Micol Avenali, Michele Giovanni Croce, Federica Ferrari, Francesca Valentino, Claudio Pacchetti, Cristina Tassorelli, Roberta Zangaglia

**Affiliations:** ^1^ Department of Brain and Behavioural Sciences University of Pavia Pavia Italy; ^2^ Headache Science and Rehabilitation Center IRCCS Mondino Foundation Pavia Italy; ^3^ Parkinson's Disease and Movement Disorders Unit IRCCS Mondino Foundation Pavia Italy; ^4^ Department of Stroke Unit and Emergency Neurology IRCCS Mondino Foundation Pavia Italy

**Keywords:** apomorphine, continuous subcutaneous infusion, CSAI, Parkinson's disease, thrombocytopenia

Continuous subcutaneous apomorphine infusion (CSAI) is a well‐tolerated treatment in Parkinson's disease (PD) with motor fluctuations.^1^ Hemolytic anemia and eosinophilic syndrome have been described as rare hematological complications.^2^, ^3^ Here we describe a case of thrombocytopenia as a likely extremely uncommon adverse event (AE) associated with apomorphine infusion in a PD patient.

A 74‐year‐old man with PD consulted for highly disabling motor fluctuations. PD onset occurred at age 52, with bradykinesia and rigidity. Optimal control of motor symptoms required numerous attempts of pharmacotherapy remodulation.

At the time of evaluation (22 years from onset) he reported prolonged *off* time with freezing of gait, frequent falls for severe levodopa‐induced dyskinesias, and anxiety as nonmotor fluctuations, affecting his quality of life. The patient was hospitalized, and CSAI was proposed. At admission, he had no relevant comorbidities and no allergies and did not receive heparin or other medication except for antiparkinsonian drugs. Peripheral blood tests at the time of admission were normal (Table S1).

CSAI by minipump was started at the rate of 1 mg/h and then gradually increased to 3.5 mg/h, for 15 hours daily. Seventy‐two hours after the first administration, the patient developed millimetric petechiae on the lower limbs, extending to the trunk and arms (Fig. 1A; Video 1). After a few hours, he showed tongue hematoma (Fig. 1B), oral petechiae, and spontaneous gingival bleeding. No major bleeding occurred.

**FIG. 1:**
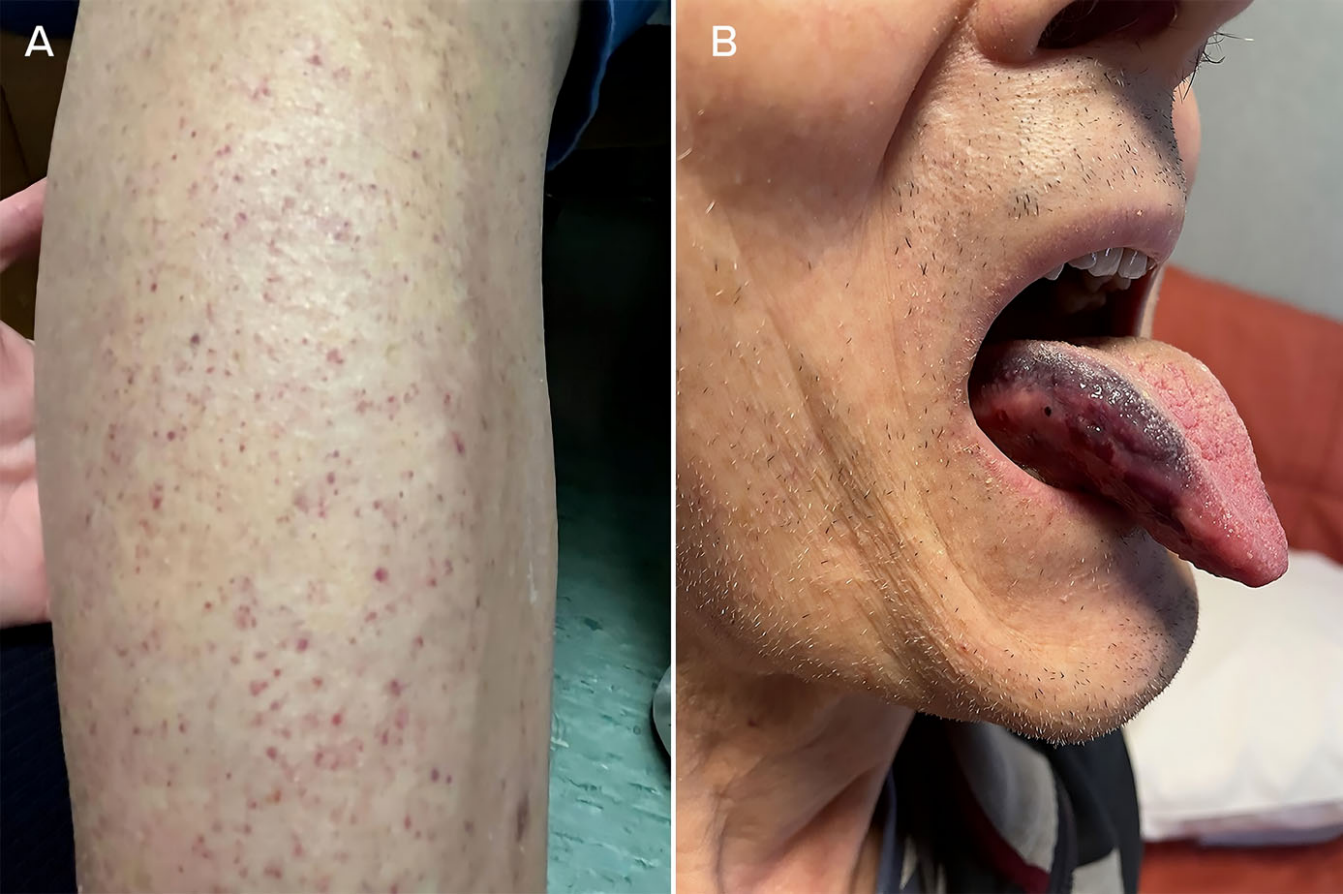
Mucocutaneous findings after 3 days from continued apomorphine subcutaneous infusion administration. (**A**) Purpura of inferior limbs. (**B**) Tongue hematoma.

**Video 1:** Purpura of inferior limbs. Note how lesions persist with finger pressure.

Repeated blood tests with citrate revealed severe thrombocytopenia (platelet count: 10.000/μL); coagulation and other hemolytic parameters were normal, including direct and indirect Coombs' test.

Suspecting an apomorphine‐related AE, the drug was immediately discontinued, and steroids were started. Platelet count gradually increased within normal range over 5 days, without transfusion. Diagnostic workup for other causes of thrombocytopenia was negative (Table S1). Cutaneous lesions progressively resolved completely in 10 days.

Drug‐induced thrombocytopenia is associated with some antibiotics and antiepileptic agents, and it usually emerges shortly after drug exposure, especially in patients previously treated with the same medication; it typically resolves within 5 to 7 days after drug discontinuation without specific treatment.

Our patient developed rapid platelet reduction after a short‐term treatment with CSAI, which completely reversed with steroid therapy after 5 days. Noteworthy, the patient was previously exposed to the drug, as “rescue” therapy for severe motor *off* phases without complications. Although we did not search for drug‐related antibodies, we hypothesize that our patient had developed anti‐apomorphine antibodies (in the framework of drug‐induced immune thrombocytopenia^4^), which, during the second exposure, quickly developed a higher binding affinity to platelets, leading to their destruction. The rapid increase in patient's platelet counts immediately after the discontinuation of apomorphine corroborates diagnostic suspicion, despite the absence of antibody testing.

To the best of our knowledge, acute thrombocytopenia occurring as a likely AE associated with apomorphine infusion does not seem to have been published so far, and it is not listed as an AE in the available literature.

In conclusion, CSAI is an effective and well‐tolerated treatment in PD patients with motor complications. Nevertheless, thrombocytopenia, as a rare hematological side effect, might occur. Neurologists should be aware of these potentially serious complications, and they should monitor patients' hemolytic parameters, especially in the first 2 to 3 days of CSAI administration and subsequently establish regular follow‐up testing.

## Author Roles

(1) Research project: A. Conception, B. Organization, C. Execution; (2) Statistical analysis: A. Design, B. Execution, C. Review and critique; (3) Manuscript: A. Writing of the first draft, B. Review and critique.

M.M.P.: 1A, 1B, 1C, 3A

M.A.: 1A, 1B, 1C, 3B

M.G.C.: 1A, 1C, 3B

F.V.: 1A, 3B

F.F.: 1A, 3B

C.T.: 1A, 3B

C.P.: 1A, 3B

R.Z.: 1A, 3B

## Disclosures

**Ethical Compliance Statement:** The authors confirm that patient informed consent was obtained for this work. The approval of an institutional review board was not required. We confirm that we have read the journal's position on issues involved in ethical publication and affirm that this work is consistent with those guidelines.

**Funding Sources and Conflicts of Interest:** The authors report no sources of funding or conflicts of interest with regard to the present topic.

**Financial Disclosures for the Previous 12 Months:** C.T. has received consulting fees or speaking honoraria from AbbVie, Dompé, Lilly, Lundbeck, Novartis, and Pfizer, and has received financial support for attending meetings from Lundbeck and Ipsen. M.A. has received consulting fees or speaking honoraria from Bial and Zambon SpA, and has received funding grants from the Ministry of Health. The remaining authors declare no sources of funding and no conflicts of interest.

## Supporting information


**Table S1.** Peripheral blood test and diagnostic workup. Baseline: at hospital admission; T1: 72 hours after CSAI (continuous subcutaneous apomorphine infusion) administration; T2: T1 + 5 days after CSAI administration; T3: T1 + 15 days. ANA, antinuclear antibodies; ANCA, antineutrophil cytoplasmic antibodies; aPL, antiphospholipid antibodies; CMV, cytomegalovirus; EBV, Epstein–Barr virus; ENA, extractable nuclear antigens; HBV, hepatitis B virus; HCV, hepatitis C virus; HIV, human immunodeficiency virus; INR, international normalized ratio; RCP, reactive protein C.
