# Supplementary material for: Apomorphine Subcutaneous Infusion Likely Induced Acute Thrombocytopenia in a Patient with Parkinson's Disease and Motor Fluctuations
Source: Mov Disord Clin Pract. 2024 Sep 27;12(1):115–7. doi: 10.1002/mdc3.14216 (PMC11736874; doi:10.1002/mdc3.14216)
Supplement: Supplementary file 1 — Table S1. Peripheral blood test and diagnostic workup. Baseline: at hospital admission; T1: 72 hours after CSAI (continuous subcutaneous apomorphine infusion) administration; T2: T1 + 5 days after CSAI administration; T3: T1 + 15 days. ANA, antinuclear antibodies; ANCA, antineutrophil cytoplasmic antibodies; aPL, antiphospholipid antibodies; CMV, cytomegalovirus; EBV, Epstein–Barr virus; ENA, extractable nuclear antigens; HBV, hepatitis B virus; HCV, hepatitis C virus; HIV, human immunodeficiency virus; INR, international normalized ratio; RCP, reactive protein C. [file MDC3-12-115-s001.docx]

**Supplementary** **Table 1: Peripheral blood test and diagnostic workup**. Baseline: at hospital admission; T1: Seventy-two hours after CSAI administration; T2: T1 + five days after CSAI administration; T3: T1 + fifteen days. INR International Normalized Ratio; RCP Reactive Protein C; HBV Hepatitis B Virus; HCV Hepatitis C Virus; HIV Human Immunodeficiency Virus; EBV Epstein-Barr Virus; CMV Cytomegalovirus; ANA Antinuclear antibodies; ENA Extractable Nuclear Antigens; ANCA Antineutrophil Cytoplasmic Antibodies; aPL Antiphospholipid antibodies.

| **Parameter**  **(normal range)** | **Baseline** | **T1** | **T2** | **T3** |
| --- | --- | --- | --- | --- |
| Hemoglobin (14-18 g/L) | 14.1 | 12.9 | 11.9 | 11.9 |
| Hematocrit (42-52%) | 42.6 | 39.5 | 35.6 | 35.8 |
| White cell count (4.800-10.800/microL) | 4.900 | 2.100 | 6.900 | 4.910 |
| Platelet count (130.000-400.000/microL) | 240.000 | 10.000; 13.000 (citrate) | 272.000 | 259.000 |
| Mean platelet volume (7.4-10.4 fl) | 9.1 | 10.4 | 8.0 | 9.9 |
| INR | 0.89 | 0.95 | - | - |
| aPTT (24.3-35 sec) | 25 | 24.6 | - | - |
| Lactate dehydrogenase (10-250 UI/L) | - | 193 | - | - |
| Haptoglobin (30-200 mg/dl) | - | 93 | - | - |
| Coombs’ test | - | Negative | - | - |
| RCP (0.0-0.5 mg/dl) | <0.1 | 1.2 |  | - |
| Viral serology including HBV, HCV, HIV, EBV, CMV | - | Negative | - | - |
| Autoimmune screening including ANA, ENA, ANCA, aPL | - | Negative | - | - |
